# A Calculus for Modular Loop Acceleration

**DOI:** 10.1007/978-3-030-45190-5_4

**Published:** 2020-03-13

**Authors:** Florian Frohn

**Affiliations:** 8 Johannes Kepler University, Linz, Austria; 9 University of Birmingham, Birmingham, UK; Max Planck Institute for Informatics, Saarland Informatics Campus, Saarbrücken, Germany

## Abstract

Loop acceleration can be used to prove safety, reachability, runtime bounds, and (non-)termination of programs operating on integers. To this end, a variety of acceleration techniques has been proposed. However, all of them are monolithic: Either they accelerate a loop successfully or they fail completely. In contrast, we present a calculus that allows for combining acceleration techniques in a modular way and we show how to integrate many existing acceleration techniques into our calculus. Moreover, we propose two novel acceleration techniques that can be incorporated into our calculus seamlessly. An empirical evaluation demonstrates the applicability of our approach.

### 1 Introduction

In the last years, loop acceleration techniques have successfully been used to build static analyses for programs operating on integers [2, 8, 11, 16–18, 28]. Essentially, such techniques extract a quantifier-free first-order formula $\psi$ from a single-path loop $\mathcal{T}$, i.e., a loop without branching in its body, such that $\psi$ under-approximates (resp. is equivalent to) $\mathcal{T}$. More specifically, each model of the resulting formula $\psi$ corresponds to an execution of $\mathcal{T}$ (and vice versa). By integrating such techniques into a suitable program-analysis framework [3, 11, 16–18, 23], whole programs can be transformed into first-order formulas which can then be analyzed by off-the-shelf solvers. Applications include proving safety [23] or reachability [23, 28], deducing bounds on the runtime complexity [16, 17], and proving (non-) termination [8, 11].

However, existing acceleration techniques only apply if certain prerequisites are in place. So the power of static analyses built upon loop acceleration depends on the applicability of the underlying acceleration technique.

In this paper, we introduce a calculus which allows for combining several acceleration techniques modularly in order to accelerate a single loop. Consequently, it can handle classes of loops where all standalone techniques fail. Moreover, we present two novel acceleration techniques and integrate them into our calculus.

In the following, we introduce preliminaries in Sec. 2. Then, we discuss existing acceleration techniques in Sec. 3. In Sec. 4, we present our calculus to combine acceleration techniques. Sec. 5 shows how existing acceleration techniques can be

* This work has been funded by DFG grant 389792660 as part of TRR 248 (see https://perspicuous-computing.science).

integrated into our framework. Next, we present two novel acceleration techniques and incorporate them into our calculus in Sec. 6. After discussing related work in Sec. 7, we demonstrate the applicability of our approach via an empirical evaluation in Sec. 8 and conclude in Sec. 9. All proofs can be found in [13].

### 2 Preliminaries

We use bold letters $\boldsymbol{x}$, $\boldsymbol{y}$, $\boldsymbol{z}$, ... for vectors. Let $\mathscr{C}(\boldsymbol{z})$ be the set of *closed-form expressions* over the variables $\boldsymbol{z}$ containing, e.g., all arithmetic expressions built from $\boldsymbol{z}$, integer constants, addition, subtraction, multiplication, division, and exponentiation.[1] We consider loops of the form

$$\textbf{while } \varphi \textbf{ do } \boldsymbol{x} \leftarrow \boldsymbol{a} \qquad (\mathcal{T}_{loop})$$

where $\boldsymbol{x}$ is a vector of $d$ pairwise different variables that range over the integers, the loop condition $\varphi \in \mathit{Prop}(\mathscr{C}(\boldsymbol{x}))$ is a finite propositional formula over the atoms $\{p > 0 \mid p \in \mathscr{C}(\boldsymbol{x})\}$, and $\boldsymbol{a} \in \mathscr{C}(\boldsymbol{x})^d$ such that the function[2] $\boldsymbol{x} \mapsto \boldsymbol{a}$ maps integers to integers. *Loop* denotes the set of all such loops.

We identify $\mathcal{T}_{loop}$ and the pair $\langle \varphi, \boldsymbol{a} \rangle$. Moreover, we identify $\boldsymbol{a}$ and the function $\boldsymbol{x} \mapsto \boldsymbol{a}$ where we sometimes write $\boldsymbol{a}(\boldsymbol{x})$ to make the variables $\boldsymbol{x}$ explicit and we use the same convention for other (vectors of) expressions. Similarly, we identify the formula $\varphi$ resp. $\varphi(\boldsymbol{x})$ and the predicate $\boldsymbol{x} \mapsto \varphi$.

Throughout this paper, let $n$ be a designated variable and let:

$$\boldsymbol{a} := \begin{pmatrix} a_1 \\ \cdots \\ a_d \end{pmatrix} \qquad \boldsymbol{x} := \begin{pmatrix} x_1 \\ \cdots \\ x_d \end{pmatrix} \qquad \boldsymbol{x}' := \begin{pmatrix} x'_1 \\ \cdots \\ x'_d \end{pmatrix} \qquad \boldsymbol{y} := \begin{pmatrix} \boldsymbol{x} \\ n \\ \boldsymbol{x}' \end{pmatrix}$$

Intuitively, the variable $n$ represents the number of loop iterations and $\boldsymbol{x}'$ corresponds to the values of the program variables $\boldsymbol{x}$ after $n$ iterations.

$\mathcal{T}_{loop}$ induces a relation $\longrightarrow_{\mathcal{T}_{loop}}$ on $\mathbb{Z}^d$:

$$\varphi(\boldsymbol{x}) \land \boldsymbol{x}' = \boldsymbol{a}(\boldsymbol{x}) \iff \boldsymbol{x} \longrightarrow_{\mathcal{T}_{loop}} \boldsymbol{x}'$$

Our goal is to find a formula $\psi \in \mathit{Prop}(\mathscr{C}(\boldsymbol{y}))$ such that

$$\psi \iff \boldsymbol{x} \longrightarrow^n_{\mathcal{T}_{loop}} \boldsymbol{x}' \qquad \text{for all } n > 0. \qquad \text{(equiv)}$$

To see why we use $\mathscr{C}(\boldsymbol{y})$ instead of, e.g., polynomials, consider the loop

$$\textbf{while } x_1 > 0 \textbf{ do } \begin{pmatrix} x_1 \\ x_2 \end{pmatrix} \leftarrow \begin{pmatrix} x_1 - 1 \\ 2 \cdot x_2 \end{pmatrix}. \qquad (\mathcal{T}_{exp})$$

Here, an acceleration technique synthesizes, e.g., the formula

$$\begin{pmatrix} x'_1 \\ x'_2 \end{pmatrix} = \begin{pmatrix} x_1 - n \\ 2^n \cdot x_2 \end{pmatrix} \land x_1 - n + 1 > 0 \qquad (\psi_{exp})$$

[1] Note that there is no widely accepted definition of "closed forms" and the results of the current paper are independent of the precise definition of $\mathscr{C}(\boldsymbol{z})$.

[2] i.e., the (anonymous) function that maps $\boldsymbol{x}$ to $\boldsymbol{a}$

where $\left(\begin{smallmatrix} x_1 - n \\ 2^n \cdot x_2 \end{smallmatrix}\right)$ is equivalent to the value of $\left(\begin{smallmatrix} x_1 \\ x_2 \end{smallmatrix}\right)$ after $n$ iterations and the inequation $x_1 - n + 1 > 0$ ensures that $\mathcal{T}_{exp}$ can be executed at least $n$ times. Clearly, the growth of $x_2$ cannot be captured by a polynomial, i.e., even the behavior of quite simple loops is beyond the expressiveness of polynomial arithmetic.

In practice, one can restrict our approach to weaker classes of expressions to ease automation, but the presented results are independent of such considerations.

Some acceleration techniques cannot guarantee (equiv), but the resulting formula is an under-approximation of $\mathcal{T}_{loop}$, i.e., we have

$$\psi \implies \boldsymbol{x} \longrightarrow^{n}_{\mathcal{T}_{loop}} \boldsymbol{x}' \qquad \text{for all } n > 0. \tag{approx}$$

If (equiv) resp. (approx) holds, then $\psi$ is *equivalent* to resp. *approximates* $\mathcal{T}_{loop}$.

**Definition 1 (Acceleration Technique).** *An* acceleration technique *is a partial function*

$$accel : Loop \rightharpoonup Prop(\mathscr{C}(\boldsymbol{y})).$$

*It is* sound *if* $accel(\mathcal{T})$ *approximates* $\mathcal{T}$ *for all* $\mathcal{T} \in \mathrm{dom}(accel)$. *It is* exact *if* $accel(\mathcal{T})$ *is equivalent to* $\mathcal{T}$ *for all* $\mathcal{T} \in \mathrm{dom}(accel)$.

### 3 Existing Acceleration Techniques

We now recall several existing acceleration techniques. In Sec. 4 we will see how these techniques can be combined in a modular way. All of them first compute a *closed form* $\boldsymbol{c} \in \mathscr{C}(\boldsymbol{x}, n)^d$ for the values of the program variables after $n$ iterations.

**Definition 2 (Closed Form).** *We call* $\boldsymbol{c} \in \mathscr{C}(\boldsymbol{x}, n)^d$ *a* closed form *of* $\mathcal{T}_{loop}$ *if*

$$\forall \boldsymbol{x} \in \mathbb{Z}^d, n \in \mathbb{N}.\ \boldsymbol{c} = \boldsymbol{a}^n(\boldsymbol{x}).$$

Here, $\boldsymbol{a}^n$ is the $n$-fold application of $\boldsymbol{a}$, i.e., $\boldsymbol{a}^0(\boldsymbol{x}) = \boldsymbol{x}$ and $\boldsymbol{a}^{n+1}(\boldsymbol{x}) = \boldsymbol{a}(\boldsymbol{a}^n(\boldsymbol{x}))$. To find closed forms, one tries to solve the system of recurrence equations $\boldsymbol{x}^{(n)} = \boldsymbol{a}(\boldsymbol{x}^{(n-1)})$ with the initial condition $\boldsymbol{x}^{(0)} = \boldsymbol{x}$. In the sequel, we assume that we can represent $\boldsymbol{a}^n(\boldsymbol{x})$ in closed form. Note that one can always do so if $\boldsymbol{a}(\boldsymbol{x}) = A\boldsymbol{x} + \boldsymbol{b}$ with $A \in \mathbb{Z}^{d \times d}$ and $\boldsymbol{b} \in \mathbb{Z}^d$, i.e., if $\boldsymbol{a}$ is affine. To this end, one considers the matrix $B := \left(\begin{smallmatrix} A & \boldsymbol{b} \\ \boldsymbol{0}^T & 1 \end{smallmatrix}\right)$ and computes its Jordan normal form $B = T^{-1}JT$ where $J$ is a block diagonal matrix (which has complex entries if $B$ has complex eigenvalues). Then the closed form for $J^n$ can be given directly (see, e.g., [31]) and $\boldsymbol{a}^n(\boldsymbol{x}) = T^{-1}J^nT\left(\begin{smallmatrix} \boldsymbol{x} \\ 1 \end{smallmatrix}\right)$. Moreover, one can compute a closed form if $\boldsymbol{a} = \left(\begin{smallmatrix} c_1 \cdot x_1 + p_1 \\ \cdots \\ c_d \cdot x_d + p_d \end{smallmatrix}\right)$ where $c_i \in \mathbb{N}$ and each $p_i$ is a polynomial over $x_1, \ldots, x_{i-1}$ [15].

#### 3.1 Acceleration via Decrease *or* Increase

The first acceleration technique discussed in this section exploits the following observation: If $\varphi(\boldsymbol{a}(\boldsymbol{x}))$ implies $\varphi(\boldsymbol{x})$ and $\varphi(\boldsymbol{a}^{n-1}(\boldsymbol{x}))$ holds, then $\mathcal{T}_{loop}$ is applicable at least $n$ times. So in other words, it requires that the indicator function (or characteristic function) $I_\varphi : \mathbb{Z}^d \to \{0, 1\}$ of $\varphi$ with $I_\varphi(\boldsymbol{x}) = 1 \iff \varphi(\boldsymbol{x})$ is monotonically decreasing w.r.t. $\boldsymbol{a}$, i.e., $I_\varphi(\boldsymbol{x}) \geq I_\varphi(\boldsymbol{a}(\boldsymbol{x}))$.

**Theorem 1 (Acceleration via Monotonic Decrease [28]).** *If*

$$\varphi(\boldsymbol{a}(\boldsymbol{x})) \implies \varphi(\boldsymbol{x}),$$

*then the following acceleration technique is exact:*

$$\mathcal{T}_{loop} \mapsto \boldsymbol{x}' = \boldsymbol{a}^n(\boldsymbol{x}) \land \varphi(\boldsymbol{a}^{n-1}(\boldsymbol{x}))$$

So for example, Thm. 1 accelerates $\mathcal{T}_{exp}$ to $\psi_{exp}$. However, the requirement $\varphi(\boldsymbol{a}(\boldsymbol{x})) \implies \varphi(\boldsymbol{x})$ is often violated in practice. To see this, consider the loop

$$\textbf{while } x_1 > 0 \land x_2 > 0 \textbf{ do } \begin{pmatrix} x_1 \\ x_2 \end{pmatrix} \leftarrow \begin{pmatrix} x_1 - 1 \\ x_2 + 1 \end{pmatrix}. \qquad (\mathcal{T}_{non\text{-}dec})$$

It cannot be accelerated with Thm. 1 as

$$x_1 - 1 > 0 \land x_2 + 1 > 0 \not\Longrightarrow x_1 > 0 \land x_2 > 0.$$

A dual acceleration technique is obtained by "reversing" the implication in the prerequisites of Thm. 1. Then $I_\varphi$ is monotonically increasing w.r.t. $\boldsymbol{a}$. So $\varphi$ is an invariant and thus $\{\boldsymbol{x} \in \mathbb{Z}^d \mid \varphi(\boldsymbol{x})\}$ is a *recurrent set* [22] of $\mathcal{T}_{loop}$.

**Theorem 2 (Acceleration via Monotonic Increase).** *If*

$$\varphi(\boldsymbol{x}) \implies \varphi(\boldsymbol{a}(\boldsymbol{x})),$$

*then the following acceleration technique is exact:*

$$\mathcal{T}_{loop} \mapsto \boldsymbol{x}' = \boldsymbol{a}^n(\boldsymbol{x}) \land \varphi(\boldsymbol{x})$$

As a minimal example, Thm. 2 accelerates

$$\textbf{while } x > 0 \textbf{ do } x \leftarrow x + 1$$

to $x' = x + n \land x > 0$.

#### 3.2 Acceleration via Decrease *and* Increase

Both acceleration techniques presented so far have been generalized in [11].

**Theorem 3 (Acceleration via Monotonicity [11]).** *If*

$$\begin{aligned} \varphi(\boldsymbol{x}) &\iff \varphi_1(\boldsymbol{x}) \land \varphi_2(\boldsymbol{x}) \land \varphi_3(\boldsymbol{x}), \\ \varphi_1(\boldsymbol{x}) &\implies \varphi_1(\boldsymbol{a}(\boldsymbol{x})), \\ \varphi_1(\boldsymbol{x}) \land \varphi_2(\boldsymbol{a}(\boldsymbol{x})) &\implies \varphi_2(\boldsymbol{x}), \qquad \textit{and} \\ \varphi_1(\boldsymbol{x}) \land \varphi_2(\boldsymbol{x}) \land \varphi_3(\boldsymbol{x}) &\implies \varphi_3(\boldsymbol{a}(\boldsymbol{x})), \end{aligned}$$

*then the following acceleration technique is exact:*

$$\mathcal{T}_{loop} \mapsto \boldsymbol{x}' = \boldsymbol{a}^n(\boldsymbol{x}) \land \varphi_1(\boldsymbol{x}) \land \varphi_2(\boldsymbol{a}^{n-1}(\boldsymbol{x})) \land \varphi_3(\boldsymbol{x})$$

Here, $\varphi_1$ and $\varphi_3$ are again invariants of the loop. Thus, as in Thm. 2 it suffices to require that they hold before entering the loop. On the other hand, $\varphi_2$ needs to satisfy a similar condition as in Thm. 1 and thus it suffices to require that $\varphi_2$ holds before the last iteration. We also say that $\varphi_2$ is a *converse invariant* (w.r.t. $\varphi_1$). It is easy to see that Thm. 3 is equivalent to Thm. 1 if $\varphi_1 \equiv \varphi_3 \equiv \top$ (where $\top$ denotes logical truth) and it is equivalent to Thm. 2 if $\varphi_2 \equiv \varphi_3 \equiv \top$.

With this approach, $\mathcal{T}_{non\text{-}dec}$ can be accelerated to

$$\begin{pmatrix} x_1' \\ x_2' \end{pmatrix} = \begin{pmatrix} x_1 - n \\ x_2 + n \end{pmatrix} \land x_2 > 0 \land x_1 - n + 1 > 0 \tag{$\psi_{non\text{-}dec}$}$$

by choosing $\varphi_1 := x_2 > 0$, $\varphi_2 := x_1 > 0$, and $\varphi_3 := \top$.

Thm. 3 naturally raises the question: Why do we need *two* invariants? To see this, consider a restriction of Thm. 3 where $\varphi_3 := \top$. It would fail for a loop like

$$\mathbf{while}\ x_1 > 0 \land x_2 > 0\ \mathbf{do}\ \begin{pmatrix} x_1 \\ x_2 \end{pmatrix} \leftarrow \begin{pmatrix} x_1 + x_2 \\ x_2 - 1 \end{pmatrix} \tag{$\mathcal{T}_{2\text{-}invs}$}$$

which can easily be handled by Thm. 3 (by choosing $\varphi_1 := \top$, $\varphi_2 := x_2 > 0$, and $\varphi_3 := x_1 > 0$). The problem is that the converse invariant $x_2 > 0$ is needed to prove invariance of $x_1 > 0$. Similarly, a restriction of Thm. 3 where $\varphi_1 := \top$ would fail for the following variant of $\mathcal{T}_{2\text{-}invs}$:

$$\mathbf{while}\ x_1 > 0 \land x_2 > 0\ \mathbf{do}\ \begin{pmatrix} x_1 \\ x_2 \end{pmatrix} \leftarrow \begin{pmatrix} x_1 - x_2 \\ x_2 + 1 \end{pmatrix}$$

Here, the problem is that the invariant $x_2 > 0$ is needed to prove converse invariance of $x_1 > 0$.

#### 3.3 Acceleration via Metering Functions

Another approach for loop acceleration uses *metering functions*, a variation of classical *ranking functions* from termination and complexity analysis [17]. While ranking functions give rise to *upper* bounds on the runtime of loops, metering functions provide *lower* runtime bounds, i.e., the definition of a metering function $mf : \mathbb{Z}^d \to \mathbb{Q}$ ensures that for each $\boldsymbol{x} \in \mathbb{Z}^d$, the loop under consideration can be applied at least $\lceil mf(\boldsymbol{x}) \rceil$ times.

**Theorem 4 (Acceleration via Metering Functions [17]).** *Let mf be a metering function for $\mathcal{T}_{loop}$. Then the following acceleration technique is sound:*

$$\mathcal{T}_{loop} \mapsto \boldsymbol{x}' = \boldsymbol{a}^n(\boldsymbol{x}) \land \varphi(\boldsymbol{x}) \land n < mf(\boldsymbol{x}) + 1$$

So using the metering function $x$, Thm. 4 accelerates $\mathcal{T}_{exp}$ to

$$\begin{pmatrix} x_1' \\ x_2' \end{pmatrix} = \begin{pmatrix} x_1 - n \\ 2^n \cdot x_2 \end{pmatrix} \land x_1 > 0 \land n < x_1 + 1 \quad \equiv \quad \psi_{exp}.$$

However, synthesizing non-trivial (i.e., non-constant) metering functions is challenging. Moreover, unless the number of iterations of $\mathcal{T}_{loop}$ equals $\lceil mf(\boldsymbol{x}) \rceil$ for all $\boldsymbol{x} \in \mathbb{Z}^d$, *acceleration via metering functions* is not exact.

*Linear* metering functions can be synthesized via Farkas' Lemma and SMT solving [17]. However, many loops do not have non-trivial linear metering functions. To see this, reconsider $\mathcal{T}_{\textit{non-dec}}$. Here, $(x_1, x_2) \mapsto x_1$ is not a metering function as $\mathcal{T}_{\textit{non-dec}}$ cannot be iterated at least $x_1$ times if $x_2 \leq 0$. Thus, [16] proposes a refinement of [17] based on metering functions of the form $\boldsymbol{x} \mapsto I_\xi(\boldsymbol{x}) \cdot f(\boldsymbol{x})$ where $\xi \in \mathit{Prop}(\mathscr{C}(\boldsymbol{x}))$ and $f$ is linear. With this improvement, the metering function $(x_1, x_2) \mapsto I_{x_2>0}(x_2) \cdot x_1$ can be used to accelerate $\mathcal{T}_{\textit{non-dec}}$ to

$$\left(\begin{smallmatrix} x_1' \\ x_2' \end{smallmatrix}\right) = \left(\begin{smallmatrix} x_1 - n \\ x_2 + n \end{smallmatrix}\right) \wedge x_1 > 0 \wedge x_2 > 0 \wedge n < x_1 + 1.$$

### 4 A Calculus for Modular Loop Acceleration

All acceleration techniques presented so far are monolithic: Either they accelerate a loop successfully or they fail completely. In other words, we cannot *combine* several techniques to accelerate a single loop. To this end, we now present a calculus that repeatedly applies acceleration techniques to simplify an *acceleration problem* resulting from a loop $\mathcal{T}_{\textit{loop}}$ until it is *solved* and hence gives rise to a suitable $\psi \in \mathit{Prop}(\mathscr{C}(\boldsymbol{y}))$ which approximates resp. is equivalent to $\mathcal{T}_{\textit{loop}}$.

**Definition 3 (Acceleration Problem).** *A tuple*

$$[\![\psi \mid \check{\varphi} \mid \widehat{\varphi} \mid \boldsymbol{a}]\!]$$

*where $\psi \in \mathit{Prop}(\mathscr{C}(\boldsymbol{y}))$, $\check{\varphi}, \widehat{\varphi} \in \mathit{Prop}(\mathscr{C}(\boldsymbol{x}))$, and $\boldsymbol{a} : \mathbb{Z}^d \to \mathbb{Z}^d$ is an* acceleration problem*. It is* consistent *if $\psi$ approximates $\langle \check{\varphi}, \boldsymbol{a} \rangle$,* exact *if $\psi$ is equivalent to $\langle \check{\varphi}, \boldsymbol{a} \rangle$, and* solved *if it is consistent and $\widehat{\varphi} \equiv \top$. The* canonical acceleration problem *of a loop $\mathcal{T}_{\textit{loop}}$ is*

$$[\![\boldsymbol{x}' = \boldsymbol{a}^n(\boldsymbol{x}) \mid \top \mid \varphi(\boldsymbol{x}) \mid \boldsymbol{a}(\boldsymbol{x})]\!] .$$

*Example 1.* The canonical acceleration problem of $\mathcal{T}_{\textit{non-dec}}$ is

$$\left[\!\!\left[ \left(\begin{smallmatrix} x_1' \\ x_2' \end{smallmatrix}\right) = \left(\begin{smallmatrix} x_1 - n \\ x_2 + n \end{smallmatrix}\right) \;\middle|\; \top \;\middle|\; x_1 > 0 \wedge x_2 > 0 \;\middle|\; \left(\begin{smallmatrix} x_1 - 1 \\ x_2 + 1 \end{smallmatrix}\right) \right]\!\!\right] .$$

The first component $\psi$ of an acceleration problem $[\![\psi \mid \check{\varphi} \mid \widehat{\varphi} \mid \boldsymbol{a}]\!]$ is the partial result that has been computed so far. The second component $\check{\varphi}$ corresponds to the part of the loop condition that has already been processed successfully. As our calculus preserves consistency, $\psi$ always approximates $\langle \check{\varphi}, \boldsymbol{a} \rangle$. The third component is the part of the loop condition that remains to be processed, i.e., the loop $\langle \widehat{\varphi}, \boldsymbol{a} \rangle$ still needs to be accelerated. The goal of our calculus is to transform a canonical into a solved acceleration problem.

More specifically, when we have simplified a canonical acceleration problem $[\![\boldsymbol{x}' = \boldsymbol{a}^n(\boldsymbol{x}) \mid \top \mid \varphi(\boldsymbol{x}) \mid \boldsymbol{a}(\boldsymbol{x})]\!]$ to $[\![\psi_1(\boldsymbol{y}) \mid \check{\varphi}(\boldsymbol{x}) \mid \widehat{\varphi}(\boldsymbol{x}) \mid \boldsymbol{a}(\boldsymbol{x})]\!]$, then $\varphi \equiv \check{\varphi} \wedge \widehat{\varphi}$ and

$$\psi_1 \implies \boldsymbol{x} \longrightarrow^n_{\langle \check{\varphi}, \boldsymbol{a} \rangle} \boldsymbol{x}'.$$

Thus, it then suffices to find some $\psi_2 \in \mathit{Prop}(\mathscr{C}(\boldsymbol{y}))$ such that

$$\boldsymbol{x} \longrightarrow^n_{\langle \check{\varphi}, \boldsymbol{a} \rangle} \boldsymbol{x}' \wedge \psi_2 \implies \boldsymbol{x} \longrightarrow^n_{\langle \widehat{\varphi}, \boldsymbol{a} \rangle} \boldsymbol{x}'. \tag{1}$$

The reason is that we have $\longrightarrow_{\langle \check{\varphi}, \boldsymbol{a} \rangle} \cap \longrightarrow_{\langle \widehat{\varphi}, \boldsymbol{a} \rangle} = \longrightarrow_{\langle \check{\varphi} \wedge \widehat{\varphi}, \boldsymbol{a} \rangle} = \longrightarrow_{\langle \varphi, \boldsymbol{a} \rangle}$ and thus

$$\psi_1 \wedge \psi_2 \implies \boldsymbol{x} \longrightarrow^n_{\langle \varphi, \boldsymbol{a} \rangle} \boldsymbol{x}',$$

i.e., $\psi_1 \wedge \psi_2$ approximates $\mathcal{T}_{loop}$.

Note that the acceleration techniques presented so far would map $\langle \widehat{\varphi}, \boldsymbol{a} \rangle$ to some $\psi_2 \in \mathit{Prop}(\mathscr{C}(\boldsymbol{y}))$ such that

$$\psi_2 \implies \boldsymbol{x} \longrightarrow^n_{\langle \widehat{\varphi}, \boldsymbol{a} \rangle} \boldsymbol{x}', \tag{2}$$

which is more restrictive than (1). In Sec. 5, we will adapt all acceleration techniques from Sec. 3 to search for some $\psi_2 \in \mathit{Prop}(\mathscr{C}(\boldsymbol{y}))$ that satisfies (1) instead of (2), i.e., we will turn them into *conditional acceleration techniques.*

**Definition 4 (Conditional Acceleration).** *We call a partial function*

$$\mathit{accel} : \mathit{Loop} \times \mathit{Prop}(\mathscr{C}(\boldsymbol{x})) \rightharpoonup \mathit{Prop}(\mathscr{C}(\boldsymbol{y})).$$

*a* conditional acceleration technique*. It is* sound *if*

$$\boldsymbol{x} \longrightarrow^n_{\langle \check{\varphi}, \boldsymbol{a} \rangle} \boldsymbol{x}' \wedge \mathit{accel}(\langle \chi, \boldsymbol{a} \rangle, \check{\varphi}) \quad \text{implies} \quad \boldsymbol{x} \longrightarrow^n_{\langle \chi, \boldsymbol{a} \rangle} \boldsymbol{x}'$$

*for all $(\langle \chi, \boldsymbol{a} \rangle, \check{\varphi}) \in \mathrm{dom}(\mathit{accel})$, $\boldsymbol{x}, \boldsymbol{x}' \in \mathbb{Z}^d$, and $n > 0$. It is* exact *if additionally*

$$\boldsymbol{x} \longrightarrow^n_{\langle \chi \wedge \check{\varphi}, \boldsymbol{a} \rangle} \boldsymbol{x}' \quad \text{implies} \quad \mathit{accel}(\langle \chi, \boldsymbol{a} \rangle, \check{\varphi})$$

*for all $(\langle \chi, \boldsymbol{a} \rangle, \check{\varphi}) \in \mathrm{dom}(\mathit{accel})$, $\boldsymbol{x}, \boldsymbol{x}' \in \mathbb{Z}^d$, and $n > 0$.*

We are now ready to present our *acceleration calculus*, which combines loop acceleration techniques in a modular way. In the following, w.l.o.g. we assume that propositional formulas are in CNF and we identify the formula $\bigwedge_{i=1}^k C_i$ with the set of clauses $\{C_i \mid 1 \leq i \leq k\}$.

**Definition 5 (Acceleration Calculus).** *The relation $\rightsquigarrow$ on acceleration problems is defined by the following rule:*

$$\frac{\emptyset \neq \chi \subseteq \widehat{\varphi} \quad \mathit{accel}(\langle \chi, \boldsymbol{a} \rangle, \check{\varphi}) = \psi_2}{[\![\psi_1 \mid \check{\varphi} \mid \widehat{\varphi} \mid \boldsymbol{a}]\!] \rightsquigarrow_{(e)} [\![\psi_1 \cup \psi_2 \mid \check{\varphi} \cup \chi \mid \widehat{\varphi} \setminus \chi \mid \boldsymbol{a}]\!]} \quad \textit{accel is a sound conditional acceleration technique}$$

*A $\rightsquigarrow$-step is* exact *(written $\rightsquigarrow_e$) if accel is exact.*

So our calculus allows us to pick a subset $\chi$ (of clauses) from the yet unprocessed condition $\widehat{\varphi}$ and "move" it to $\check{\varphi}$, which has already been processed successfully. To this end, $\langle \chi, \boldsymbol{a} \rangle$ needs to be accelerated by a conditional acceleration technique, i.e., when accelerating $\langle \chi, \boldsymbol{a} \rangle$ we may assume $\boldsymbol{x} \longrightarrow^n_{\langle \check{\varphi}, \boldsymbol{a} \rangle} \boldsymbol{x}'$.

Note that every acceleration technique trivially gives rise to a conditional acceleration technique (by disregarding the second argument $\check{\varphi}$ of *accel* in Def. 4). Thus, our calculus allows for combining arbitrary existing acceleration techniques without adapting them. However, many acceleration techniques can easily be turned into more sophisticated conditional acceleration techniques (cf. Sec. 5), which increases the power of our approach.

*Example 2.* We continue Ex. 1 and fix $\chi := x_1 > 0$. Thus, we need to accelerate the loop $\langle x_1 > 0, \left(\begin{smallmatrix} x_1-1 \\ x_2+1 \end{smallmatrix}\right)\rangle$ to enable a $\rightsquigarrow$-step. We obtain

$$\begin{aligned}
& \left[\!\!\left[ \psi^{init}_{non\text{-}dec} := \begin{pmatrix} x_1' \\ x_2' \end{pmatrix} = \left(\begin{smallmatrix} x_1-n \\ x_2+n \end{smallmatrix}\right) \;\middle|\; \top \;\middle|\; x_1 > 0 \land x_2 > 0 \;\middle|\; \left(\begin{smallmatrix} x_1-1 \\ x_2+1 \end{smallmatrix}\right) \right]\!\!\right] \\
\overset{Thm.\ 1}{\rightsquigarrow_e}\ & [\![ \psi^{init}_{non\text{-}dec} \land x_1 - n + 1 > 0 \mid x_1 > 0 \mid x_2 > 0 \mid \left(\begin{smallmatrix} x_1-1 \\ x_2+1 \end{smallmatrix}\right) ]\!] \\
\overset{Thm.\ 2}{\rightsquigarrow_e}\ & [\![ \psi^{init}_{non\text{-}dec} \land x_1 - n + 1 > 0 \land x_2 > 0 \mid x_1 > 0 \land x_2 > 0 \mid \top \mid \left(\begin{smallmatrix} x_1-1 \\ x_2+1 \end{smallmatrix}\right) ]\!] \\
=\ & [\![ \psi_{non\text{-}dec} \mid x_1 > 0 \land x_2 > 0 \mid \top \mid \left(\begin{smallmatrix} x_1-1 \\ x_2+1 \end{smallmatrix}\right) ]\!]
\end{aligned}$$

where Thm. 2 was applied to the loop $\langle x_2 > 0, \left(\begin{smallmatrix} x_1-1 \\ x_2+1 \end{smallmatrix}\right)\rangle$ in the second step. Thus, we successfully constructed the formula $\psi_{non\text{-}dec}$, which is equivalent to $\mathcal{T}_{non\text{-}dec}$.

The crucial property of our calculus is the following.

**Lemma 1.** *$\rightsquigarrow$ preserves consistency and $\rightsquigarrow_e$ preserves exactness.*

Then the correctness of our calculus follows immediately. The reason is that $[\![\boldsymbol{x}' = \boldsymbol{a}^n(\boldsymbol{x}) \mid \top \mid \varphi(\boldsymbol{x}) \mid \boldsymbol{a}(\boldsymbol{x})]\!] \rightsquigarrow^*_{(e)} [\![\psi(\boldsymbol{y}) \mid \check{\varphi}(\boldsymbol{x}) \mid \top \mid \boldsymbol{a}(\boldsymbol{x})]\!]$ implies $\varphi \equiv \check{\varphi}$.

**Theorem 5 (Correctness of $\rightsquigarrow$).** *If*

$$[\![\boldsymbol{x}' = \boldsymbol{a}^n(\boldsymbol{x}) \mid \top \mid \varphi(\boldsymbol{x}) \mid \boldsymbol{a}(\boldsymbol{x})]\!] \rightsquigarrow^* [\![\psi(\boldsymbol{y}) \mid \check{\varphi}(\boldsymbol{x}) \mid \top \mid \boldsymbol{a}(\boldsymbol{x})]\!],$$

*then $\psi$ approximates $\mathcal{T}_{loop}$. If*

$$[\![\boldsymbol{x}' = \boldsymbol{a}^n(\boldsymbol{x}) \mid \top \mid \varphi(\boldsymbol{x}) \mid \boldsymbol{a}(\boldsymbol{x})]\!] \rightsquigarrow^*_e [\![\psi(\boldsymbol{y}) \mid \check{\varphi}(\boldsymbol{x}) \mid \top \mid \boldsymbol{a}(\boldsymbol{x})]\!],$$

*then $\psi$ is equivalent to $\mathcal{T}_{loop}$.*

Termination of our calculus is trivial, as the size of the third component $\widehat{\varphi}$ of the acceleration problem is decreasing.

**Theorem 6 (Termination of $\rightsquigarrow$).** *$\rightsquigarrow$ terminates.*

### 5 Conditional Acceleration Techniques

We now show how to turn the acceleration techniques from Sec. 3 into conditional acceleration techniques, starting with *acceleration via monotonic decrease*.

**Theorem 7 (Conditional Acceleration via Monotonic Decrease).** *If*

$$\check{\varphi}(\boldsymbol{x}) \land \chi(\boldsymbol{a}(\boldsymbol{x})) \implies \chi(\boldsymbol{x}),$$

*then the following conditional acceleration technique is exact:*

$$(\langle \chi, \boldsymbol{a} \rangle, \check{\varphi}) \mapsto \boldsymbol{x}' = \boldsymbol{a}^n(\boldsymbol{x}) \land \chi(\boldsymbol{a}^{n-1}(\boldsymbol{x}))$$

So we just add $\check{\varphi}$ to the premise of the implication that needs to be checked to apply *acceleration via monotonic decrease*. Thm. 2 can be adapted analogously.

**Theorem 8 (Conditional Acceleration via Monotonic Increase).** *If*

$$\breve{\varphi}(\boldsymbol{x}) \wedge \chi(\boldsymbol{x}) \implies \chi(\boldsymbol{a}(\boldsymbol{x})),$$

*then the following conditional acceleration technique is exact:*

$$(\langle \chi, \boldsymbol{a} \rangle, \breve{\varphi}) \mapsto \boldsymbol{x}' = \boldsymbol{a}^n(\boldsymbol{x}) \wedge \chi(\boldsymbol{x})$$

*Example 3.* For the canonical acceleration problem of $\mathcal{T}_{\mathit{2\text{-}invs}}$, we obtain:

$$\begin{aligned} & \llbracket \boldsymbol{x}' = \boldsymbol{a}^n_{\mathit{2\text{-}invs}}(\boldsymbol{x}) \mid \top \mid x_1 > 0 \wedge x_2 > 0 \mid \boldsymbol{a}_{\mathit{2\text{-}invs}} := \left(\begin{smallmatrix} x_1 + x_2 \\ x_2 - 1 \end{smallmatrix}\right) \rrbracket \\ \overset{\text{Thm. 7}}{\rightsquigarrow_e} \; & \llbracket \boldsymbol{x}' = \boldsymbol{a}^n_{\mathit{2\text{-}invs}}(\boldsymbol{x}) \wedge x_2 - n + 1 > 0 \mid x_2 > 0 \mid x_1 > 0 \mid \boldsymbol{a}_{\mathit{2\text{-}invs}} \rrbracket \\ \overset{\text{Thm. 8}}{\rightsquigarrow_e} \; & \llbracket \boldsymbol{x}' = \boldsymbol{a}^n_{\mathit{2\text{-}invs}}(\boldsymbol{x}) \wedge x_2 - n + 1 > 0 \wedge x_1 > 0 \mid x_2 > 0 \wedge x_1 > 0 \mid \top \mid \boldsymbol{a}_{\mathit{2\text{-}invs}} \rrbracket \end{aligned}$$

While we could also use Thm. 1 for the first step, Thm. 2 is inapplicable in the second step. The reason is that we need the converse invariant $x_2 > 0$ to prove invariance of $x_1 > 0$.

It is not a coincidence that $\mathcal{T}_{\mathit{2\text{-}invs}}$, which could also be accelerated with *acceleration via monotonicity* (cf. Thm. 3) directly, can be handled by applying our novel calculus with Theorems 7 and 8.

*Remark 1.* If applying *acceleration via monotonicity* to $\mathcal{T}_{\mathit{loop}}$ yields $\psi$, then

$$\llbracket \boldsymbol{x}' = \boldsymbol{a}^n(\boldsymbol{x}) \mid \top \mid \varphi(\boldsymbol{x}) \mid \boldsymbol{a}(\boldsymbol{x}) \rrbracket \rightsquigarrow_e^{\leq 3} \llbracket \psi(\boldsymbol{y}) \mid \varphi(\boldsymbol{x}) \mid \top \mid \boldsymbol{a}(\boldsymbol{x}) \rrbracket$$

where either Thm. 7 or Thm. 8 is applied in each $\rightsquigarrow_e$-step.

Thus, there is no need for a conditional variant of *acceleration via monotonicity*. Note that combining Theorems 7 and 8 with our calculus is also useful for loops where *acceleration via monotonicity* is inapplicable.

*Example 4.* Consider the following loop, which can be accelerated by splitting its guard into one invariant and two converse invariants.

$$\textbf{while } x_1 > 0 \wedge x_2 > 0 \wedge x_3 > 0 \textbf{ do } \begin{pmatrix} x_1 \\ x_2 \\ x_3 \end{pmatrix} \leftarrow \begin{pmatrix} x_1 - 1 \\ x_2 + x_1 \\ x_3 - x_2 \end{pmatrix} \qquad (\mathcal{T}_{\mathit{2\text{-}c\text{-}invs}})$$

Let

$$\begin{aligned} \varphi_{\mathit{2\text{-}c\text{-}invs}} &:= x_1 > 0 \wedge x_2 > 0 \wedge x_3 > 0, \\ \boldsymbol{a}_{\mathit{2\text{-}c\text{-}invs}} &:= \begin{pmatrix} x_1 - 1 \\ x_2 + x_1 \\ x_3 - x_2 \end{pmatrix}, \\ \psi^{\mathit{init}}_{\mathit{2\text{-}c\text{-}invs}} &:= \boldsymbol{x}' = \boldsymbol{a}^n_{\mathit{2\text{-}c\text{-}invs}}(\boldsymbol{x}), \end{aligned}$$

and let $x_i^{(m)}$ be the $i^{th}$ component of $\boldsymbol{a}^m_{\mathit{2\text{-}c\text{-}invs}}(\boldsymbol{x})$. Starting with the canonical acceleration problem of $\mathcal{T}_{\mathit{2\text{-}c\text{-}invs}}$, we obtain:

$$\begin{aligned} & \left\llbracket \psi^{\mathit{init}}_{\mathit{2\text{-}c\text{-}invs}} \;\middle|\; \top \;\middle|\; \varphi_{\mathit{2\text{-}c\text{-}invs}} \;\middle|\; \boldsymbol{a}_{\mathit{2\text{-}c\text{-}invs}} \right\rrbracket \\ \overset{\text{Thm. 7}}{\rightsquigarrow_e} \; & \left\llbracket \psi^{\mathit{init}}_{\mathit{2\text{-}c\text{-}invs}} \wedge x_1^{(n-1)} > 0 \;\middle|\; x_1 > 0 \;\middle|\; x_2 > 0 \wedge x_3 > 0 \;\middle|\; \boldsymbol{a}_{\mathit{2\text{-}c\text{-}invs}} \right\rrbracket \\ \overset{\text{Thm. 8}}{\rightsquigarrow_e} \; & \left\llbracket \psi^{\mathit{init}}_{\mathit{2\text{-}c\text{-}invs}} \wedge x_1^{(n-1)} > 0 \wedge x_2 > 0 \;\middle|\; x_1 > 0 \wedge x_2 > 0 \;\middle|\; x_3 > 0 \;\middle|\; \boldsymbol{a}_{\mathit{2\text{-}c\text{-}invs}} \right\rrbracket \\ \overset{\text{Thm. 7}}{\rightsquigarrow_e} \; & \left\llbracket \psi^{\mathit{init}}_{\mathit{2\text{-}c\text{-}invs}} \wedge x_1^{(n-1)} > 0 \wedge x_2 > 0 \wedge x_3^{(n-1)} > 0 \;\middle|\; \varphi_{\mathit{2\text{-}c\text{-}invs}} \;\middle|\; \top \;\middle|\; \boldsymbol{a}_{\mathit{2\text{-}c\text{-}invs}} \right\rrbracket \end{aligned}$$

Finally, we present a variant of Thm. 4 for conditional acceleration. The idea is similar to the approach for deducing metering functions of the form $\boldsymbol{x} \mapsto I_{\check{\varphi}}(\boldsymbol{x}) \cdot f(\boldsymbol{x})$ from [16] (see Sec. 3.3 for details). But in contrast to [16], in our setting the "conditional" part $\check{\varphi}$ does not need to be an invariant of the loop.

**Theorem 9 (Conditional Acceleration via Metering Functions).** *Let $mf : \mathbb{Z}^d \to \mathbb{Q}$. If*

$$\begin{aligned} \check{\varphi}(\boldsymbol{x}) \wedge \quad \chi(\boldsymbol{x}) &\implies mf(\boldsymbol{x}) - mf(\boldsymbol{a}(\boldsymbol{x})) \leq 1 \qquad \text{and} \\ \check{\varphi}(\boldsymbol{x}) \wedge \neg\chi(\boldsymbol{x}) &\implies mf(\boldsymbol{x}) \leq 0, \end{aligned}$$

*then the following conditional acceleration technique is sound:*

$$(\langle \chi, \boldsymbol{a} \rangle, \check{\varphi}) \mapsto \boldsymbol{x}' = \boldsymbol{a}^n(\boldsymbol{x}) \wedge \chi(\boldsymbol{x}) \wedge n < mf(\boldsymbol{x}) + 1$$

## 6 Acceleration via Eventual Monotonicity

The combination of the calculus from Sec. 4 and the conditional acceleration techniques from Sec. 5 still fails to handle certain interesting classes of loops. Thus, to improve the applicability of our approach we now present two new acceleration techniques based on *eventual* monotonicity.

### 6.1 Acceleration via Eventual Decrease

All (combinations of) techniques presented so far fail for the following example.

$$\textbf{while } x_1 > 0 \textbf{ do } \begin{pmatrix} x_1 \\ x_2 \end{pmatrix} \leftarrow \begin{pmatrix} x_1 + x_2 \\ x_2 - 1 \end{pmatrix} \qquad (\mathcal{T}_{ev\text{-}dec})$$

The reason is that $x_1$ does not behave monotonically, i.e., $x_1 > 0$ is neither an invariant nor a converse invariant. Essentially, $\mathcal{T}_{ev\text{-}dec}$ proceeds in two phases: In the first (optional) phase, $x_2$ is positive and hence the value of $x_1$ is monotonically increasing. In the second phase, $x_2$ is non-positive and consequently the value of $x_1$ decreases (weakly) monotonically. The crucial observation is that once the value of $x_1$ decreases, it can never increase again. Thus, despite the non-monotonic behavior of $x_1$, it suffices to require that $x_1 > 0$ holds before the first and before the $n^{th}$ loop iteration to ensure that the loop can be iterated at least $n$ times.

**Theorem 10 (Acceleration via Eventual Decrease).** *If $\varphi(\boldsymbol{x}) \equiv \bigwedge_{i=1}^{k} C_i$ where each $C_i$ contains an inequation $expr_i(\boldsymbol{x}) > 0$ such that*

$$expr_i(\boldsymbol{x}) \geq expr_i(\boldsymbol{a}(\boldsymbol{x})) \implies expr_i(\boldsymbol{a}(\boldsymbol{x})) \geq expr_i(\boldsymbol{a}^2(\boldsymbol{x})),$$

*then the following acceleration technique is sound:*

$$\mathcal{T}_{loop} \mapsto \boldsymbol{x}' = \boldsymbol{a}^n(\boldsymbol{x}) \wedge \bigwedge_{i=1}^{k} \left( expr_i(\boldsymbol{x}) > 0 \wedge expr_i(\boldsymbol{a}^{n-1}(\boldsymbol{x})) > 0 \right)$$

*If $C_i \equiv expr_i > 0$ for all $i \in [1, k]$, then it is exact.*

With Thm. 10, we can accelerate $\mathcal{T}_{ev\text{-}dec}$ to

$$\begin{pmatrix} x_1' \\ x_2' \end{pmatrix} = \begin{pmatrix} \frac{n-n^2}{2} + x_2 \cdot n + x_1 \\ x_2 - n \end{pmatrix} \land x_1 > 0 \land \frac{n-1-(n-1)^2}{2} + x_2 \cdot (n-1) + x_1 > 0$$

as we have

$$(x_1 \geq x_1 + x_2) \equiv (0 \geq x_2) \implies (0 \geq x_2 - 1) \equiv (x_1 + x_2 \geq x_1 + x_2 + x_2 - 1).$$

Turning Thm. 10 into a conditional acceleration technique is straightforward.

**Theorem 11 (Conditional Acceleration via Eventual Decrease).** *If we have $\chi(\boldsymbol{x}) \equiv \bigwedge_{i=1}^{k} C_i$ where each $C_i$ contains an inequation $expr_i(\boldsymbol{x}) > 0$ such that*

$$\breve{\varphi}(\boldsymbol{x}) \land expr_i(\boldsymbol{x}) \geq expr_i(\boldsymbol{a}(\boldsymbol{x})) \implies expr_i(\boldsymbol{a}(\boldsymbol{x})) \geq expr_i(\boldsymbol{a}^2(\boldsymbol{x})), \qquad (3)$$

*then the following conditional acceleration technique is sound:*

$$(\langle \chi, \boldsymbol{a} \rangle, \breve{\varphi}) \mapsto \boldsymbol{x}' = \boldsymbol{a}^n(\boldsymbol{x}) \land \bigwedge_{i=1}^{k} \left( expr_i(\boldsymbol{x}) > 0 \land expr_i(\boldsymbol{a}^{n-1}(\boldsymbol{x})) > 0 \right)$$

*If $C_i \equiv expr_i > 0$ for all $i \in [1, k]$, then it is exact.*

*Example 5.* Consider the following variant of $\mathcal{T}_{ev\text{-}dec}$.

$$\textbf{while } x_1 > 0 \land x_3 > 0 \textbf{ do } \begin{pmatrix} x_1 \\ x_2 \\ x_3 \end{pmatrix} \leftarrow \begin{pmatrix} x_1 + x_2 \\ x_2 - x_3 \\ x_3 \end{pmatrix}$$

Starting with its canonical acceleration problem, we get

$$\left[\!\left[ \boldsymbol{x}' = \boldsymbol{a}^n(\boldsymbol{x}) \;\middle|\; \top \;\middle|\; x_1 > 0 \land x_3 > 0 \;\middle|\; \boldsymbol{a} := \begin{pmatrix} x_1 + x_2 \\ x_2 - x_3 \\ x_3 \end{pmatrix} \right]\!\right]$$

$$\overset{Thm.\ 8}{\leadsto_e} [\![ \boldsymbol{x}' = \boldsymbol{a}^n(\boldsymbol{x}) \land x_3 > 0 \mid x_3 > 0 \mid x_1 > 0 \mid \boldsymbol{a} ]\!]$$

$$\overset{Thm.\ 11}{\leadsto_e} \left[\!\left[ \boldsymbol{x}' = \boldsymbol{a}^n(\boldsymbol{x}) \land x_3 > 0 \land x_1 > 0 \land x_1^{(n-1)} > 0 \;\middle|\; x_3 > 0 \land x_1 > 0 \;\middle|\; \top \;\middle|\; \boldsymbol{a} \right]\!\right]$$

where the second step can be performed via Thm. 11 as

$$(\breve{\varphi}(\boldsymbol{x}) \land expr(\boldsymbol{x}) \geq expr(\boldsymbol{a}(\boldsymbol{x}))) \equiv (x_3 > 0 \land x_1 \geq x_1 + x_2) \equiv (x_3 > 0 \land 0 \geq x_2)$$

implies

$$(0 \geq x_2 - x_3) \equiv (x_1 + x_2 \geq x_1 + x_2 + x_2 - x_3) \equiv (expr(\boldsymbol{a}(\boldsymbol{x})) \geq expr(\boldsymbol{a}^2(\boldsymbol{x}))).$$

#### 6.2 Acceleration via Eventual Increase

Still, all (combinations of) techniques presented so far fail for

$$\textbf{while } x_1 > 0 \textbf{ do } \begin{pmatrix} x_1 \\ x_2 \end{pmatrix} \leftarrow \begin{pmatrix} x_1 + x_2 \\ x_2 + 1 \end{pmatrix}. \qquad (\mathcal{T}_{ev\text{-}inc})$$

As in the case of $\mathcal{T}_{ev\text{-}dec}$, the value of $x_1$ does not behave monotonically, i.e., $x_1 > 0$ is neither an invariant nor a converse invariant. However, this time $x_1$ is eventually *increasing*, i.e., once $x_1$ starts to grow, it never decreases again. Thus, in this case it suffices to require that $x_1$ is positive and (weakly) increasing.

**Theorem 12 (Acceleration via Eventual Increase).** *If* $\varphi(\boldsymbol{x}) \equiv \bigwedge_{i=1}^{k} C_i$ *where each* $C_i$ *contains an inequation* $expr_i(\boldsymbol{x}) > 0$ *such that*

$$expr_i(\boldsymbol{x}) \leq expr_i(\boldsymbol{a}(\boldsymbol{x})) \implies expr_i(\boldsymbol{a}(\boldsymbol{x})) \leq expr_i(\boldsymbol{a}^2(\boldsymbol{x})),$$

*then the following acceleration technique is sound:*

$$\mathcal{T}_{loop} \mapsto \boldsymbol{x}' = \boldsymbol{a}^n(\boldsymbol{x}) \wedge \bigwedge_{i=1}^{k} 0 < expr_i(\boldsymbol{x}) \leq expr_i(\boldsymbol{a}(\boldsymbol{x}))$$

With Thm. 12, we can accelerate $\mathcal{T}_{ev\text{-}inc}$ to

$$\begin{pmatrix} x_1' \\ x_2' \end{pmatrix} = \begin{pmatrix} \frac{n^2-n}{2} + x_2 \cdot n + x_1 \\ x_2 + n \end{pmatrix} \wedge 0 < x_1 \leq x_1 + x_2 \qquad (\psi_{ev\text{-}inc})$$

as we have

$$(x_1 \leq x_1 + x_2) \equiv (0 \leq x_2) \implies (0 \leq x_2 + 1) \equiv (x_1 + x_2 \leq x_1 + x_2 + x_2 + 1).$$

However, Thm. 12 is *not* exact, as the resulting formula only covers program runs where each $expr_i$ behaves monotonically. So $\psi_{ev\text{-}inc}$ only covers those runs of $\mathcal{T}_{ev\text{-}inc}$ where the initial value of $x_2$ is non-negative. Again, turning Thm. 12 into a conditional acceleration technique is straightforward.

**Theorem 13 (Conditional Acceleration via Eventual Increase).** *If we have* $\chi(\boldsymbol{x}) \equiv \bigwedge_{i=1}^{k} C_i$ *where each* $C_i$ *contains an inequation* $expr_i(\boldsymbol{x}) > 0$ *such that*

$$\breve{\varphi}(\boldsymbol{x}) \wedge expr_i(\boldsymbol{x}) \leq expr_i(\boldsymbol{a}(\boldsymbol{x})) \implies expr_i(\boldsymbol{a}(\boldsymbol{x})) \leq expr_i(\boldsymbol{a}^2(\boldsymbol{x})), \qquad (4)$$

*then the following conditional acceleration technique is sound:*

$$(\langle \chi, \boldsymbol{a} \rangle, \breve{\varphi}) \mapsto \boldsymbol{x}' = \boldsymbol{a}^n(\boldsymbol{x}) \wedge \bigwedge_{i=1}^{k} 0 < expr_i(\boldsymbol{x}) \leq expr_i(\boldsymbol{a}(\boldsymbol{x}))$$

*Example 6.* Consider the following variant of $\mathcal{T}_{ev\text{-}inc}$.

$$\textbf{while } x_1 > 0 \wedge x_3 > 0 \textbf{ do } \begin{pmatrix} x_1 \\ x_2 \\ x_3 \end{pmatrix} \leftarrow \begin{pmatrix} x_1 + x_2 \\ x_2 + x_3 \\ x_3 \end{pmatrix}$$

Starting with its canonical acceleration problem, we get

$$\begin{aligned} & \left[\!\!\left[ \boldsymbol{x}' = \boldsymbol{a}^n(\boldsymbol{x}) \;\middle|\; \top \;\middle|\; x_1 > 0 \wedge x_3 > 0 \;\middle|\; \boldsymbol{a} := \begin{pmatrix} x_1 + x_2 \\ x_2 + x_3 \\ x_3 \end{pmatrix} \right]\!\!\right] \\ \overset{Thm.\ 8}{\rightsquigarrow_e}\ & [\![ \boldsymbol{x}' = \boldsymbol{a}^n(\boldsymbol{x}) \wedge x_3 > 0 \mid x_3 > 0 \mid x_1 > 0 \mid \boldsymbol{a} ]\!] \\ \overset{Thm.\ 13}{\rightsquigarrow}\ & [\![ \boldsymbol{x}' = \boldsymbol{a}^n(\boldsymbol{x}) \wedge x_3 > 0 \wedge 0 < x_1 \leq x_1 + x_2 \mid x_3 > 0 \wedge x_1 > 0 \mid \top \mid \boldsymbol{a} ]\!] \end{aligned}$$

where the second step can be performed via Thm. 13 as

$$(\breve{\varphi}(\boldsymbol{x}) \wedge expr(\boldsymbol{x}) \leq expr(\boldsymbol{a}(\boldsymbol{x}))) \equiv (x_3 > 0 \wedge x_1 \leq x_1 + x_2) \equiv (x_3 > 0 \wedge 0 \leq x_2)$$

implies

$$(0 \leq x_2 + x_3) \equiv (x_1 + x_2 \leq x_1 + x_2 + x_2 + x_3) \equiv (expr(\boldsymbol{a}(\boldsymbol{x})) \leq expr(\boldsymbol{a}^2(\boldsymbol{x}))).$$

We also considered versions of Theorems 11 and 13 where the inequations in (3) resp. (4) are strict, but this did not lead to an improvement in our experiments. Moreover, we experimented with a variant of Thm. 13 that splits the loop under consideration into two consecutive loops, accelerates them independently, and composes the results. While such an approach can accelerate loops like $\psi_{ev\text{-}inc}$ exactly, the impact on our experimental results was minimal. Thus, we postpone an in-depth investigation of this idea to future work.

### 7 Related Work

Acceleration-like techniques are also used in *over-approximating* settings (see, e.g., [10, 20, 21, 25, 26, 29, 32, 33]), whereas we consider *exact* and *under-approximating* loop acceleration techniques. As many related approaches have already been discussed in Sec. 3, we only mention two more techniques here.

First, [4, 7] presents an exact acceleration technique for *finite monoid affine transformations* (FMATs), i.e., loops with linear arithmetic whose body is of the form $\boldsymbol{x} \leftarrow A\boldsymbol{x} + \boldsymbol{b}$ where $\{A^i \mid i \in \mathbb{N}\}$ is finite. For such loops, Presburger-Arithmetic is sufficient to construct an equivalent formula $\psi$, i.e., it can be expressed in a decidable logic. In general, this is clearly not the case for the techniques presented in the current paper (which may even synthesize non-polynomial closed forms, see $\mathcal{T}_{exp}$). As a consequence and in contrast to our technique, this approach cannot handle loops where the values of variables grow super-linearly (i.e., it cannot handle examples like $\mathcal{T}_{2\text{-}invs}$). Implementations are available in the tools FAST [2] and Flata [24]. Further theoretical results on linear transformations whose $n$-fold closure is definable in (extensions of) Presburger-Arithmetic can be found in [5].

Second, [6] shows that *octagonal relations* can be accelerated exactly and in [27], it is proven that such relations can even be accelerated in polynomial time. This generalizes earlier results for *difference bound constraints* [9]. As in the case of FMATs, the resulting formula can be expressed in Presburger-Arithmetic. Octagonal relations are defined by a finite conjunction $\xi$ of inequations of the form $\pm x \pm y \leq c$, $x, y \in \boldsymbol{x} \cup \boldsymbol{x}'$, $c \in \mathbb{Z}$. Then $\xi$ induces the relation $\boldsymbol{x} \longrightarrow_{\xi} \boldsymbol{x}' \iff \xi(\boldsymbol{x}, \boldsymbol{x}')$. So in contrast to the loops considered in the current paper where $\boldsymbol{x}'$ is uniquely determined by $\boldsymbol{x}$, octagonal relations can represent non-deterministic programs. Therefore and due to the restricted form of octagonal relations, the work from [6, 27] is orthogonal to ours.

### 8 Implementation and Experiments

We prototypically implemented our approach in our open-source Loop Acceleration Tool LoAT [11, 16, 17]:

https://github.com/aprove-developers/LoAT/tree/tacas20

It uses Z3 [30] to check implications and PURRS [1] to compute closed forms.

For technical reasons, the closed forms computed by LoAT are valid only if $n > 0$, whereas Def. 2 requires them to be valid for all $n \in \mathbb{N}$. The reason is that PURRS has only limited support for initial conditions. In the future, we plan to use a different recurrence solver to circumvent this problem. Thus, LoAT's results are only correct for all $n > 1$ (instead of all $n > 0$). Moreover, LoAT can currently compute closed forms only if the loop body is *triangular*, meaning that each $a_i$ is an expression over $x_1, \ldots, x_i$. The reason is that PURRS cannot solve *systems* of recurrence equations, but only a single recurrence equation at a time. However, LoAT failed to compute closed forms for just 26 out of 1511 loops in our experiments, i.e., this appears to be a minor restriction in practice. Furthermore, *conditional acceleration via metering functions* has not yet been integrated into the implementation of our calculus. While LoAT can synthesize formulas with non-polynomial arithmetic, it cannot yet parse them, i.e., the input is restricted to polynomials. Finally, LoAT does not yet support disjunctive loop conditions.

Apart from these differences, our implementation closely follows the current paper. It repeatedly applies the conditional acceleration techniques from Sections 5 and 6 with the following priorities: *Thm.* 8 $>$ *Thm.* 7 $>$ *Thm.* 11 $>$ *Thm.* 13.

To evaluate our approach, we extracted 1511 loops with conjunctive guards from the category *Termination of Integer Transition Systems* of the *Termination Problems Database* [35], the benchmark collection which is used at the annual *Termination and Complexity Competition* [19], as follows:

1. We parsed all examples with LoAT and extracted each single-path loop with conjunctive guard (resulting in 3829 benchmarks).
2. We removed duplicates by checking syntactic equality (resulting in 2825 benchmarks).
3. We removed loops whose runtime is trivially constant using an incomplete check (resulting in 1733 benchmarks).
4. We removed loops which do not admit any terminating runs, i.e., loops where Thm. 2 applies (resulting in 1511 benchmarks).

We compared our implementation with LoAT's implementation of *acceleration via monotonicity* (Thm. 3, [11]) and its implementation of *acceleration via metering functions* (Thm. 4, [17]), which also incorporates the improvements proposed in [16]. We did not include the techniques from Theorems 1 and 2 in our evaluation, as they are subsumed by *acceleration via monotonicity*. Furthermore, we compared with Flata [24], which implements the techniques to accelerate FMATs and octagonal relations discussed in Sec. 7. Note that our benchmark collection contains 16 loops with non-linear arithmetic where Flata is bound to fail, since it only supports linear arithmetic. We did not compare with FAST [2], which uses a similar approach as the more recent tool Flata.

All tests have been run on StarExec [34]. The results can be seen in Table 1. They show that our novel calculus was superior to the competing techniques in our experiments. In all but 7 cases where our calculus successfully accelerated the given loop, the resulting formula was polynomial. Thus, integrating our approach into existing acceleration-based verification techniques should not present major obstacles w.r.t. automation.

| | LoAT | Monot. | Meter | Flata |
|---|---|---|---|---|
| exact | 1444 | 845 | 0[3] | 1231 |
| approx | 38 | 0 | 733 | 0 |
| fail | 29 | 666 | 778 | 280 |
| avg rt | 0.16s | 0.11s | 0.09s | 0.47s |

**Table 1.**

| | ~~Ev-Inc~~ | ~~Ev-Dec~~ | ~~Ev-Mon~~ |
|---|---|---|---|
| exact | 1444 | 845 | 845 |
| approx | 0 | 493 | 0 |
| fail | 67 | 173 | 666 |
| avg rt | 0.15s | 0.14s | 0.09s |

**Table 2.**

LoAT: Acceleration calculus + Theorems 7, 8, 11 and 13
Monot.: *Acceleration via Monotonicity*, Thm. 3
Meter: *Acceleration via Metering Functions*, Thm. 4
Flata: The tool Flata, see http://nts.imag.fr/index.php/Flata
~~Ev-Inc~~: Acceleration calculus + Theorems 7, 8 and 11
~~Ev-Dec~~: Acceleration calculus + Theorems 7, 8 and 13
~~Ev-Mon~~: Acceleration calculus + Theorems 7 and 8
exact: Number of examples that were accelerated *exactly*
approx: Number of examples that were accelerated *approximately*
fail: Number of examples that could not be accelerated
avg rt: Average runtime per example

Furthermore, we evaluated the impact of our new acceleration techniques from Sec. 6 independently. To this end, we once disabled *acceleration via eventual increase*, *acceleration via eventual decrease*, and both of them. The results can be seen in Table 2. They show that our calculus does not improve over *acceleration via monotonicity* if both *acceleration via eventual increase* and *acceleration via eventual decrease* are disabled (i.e., our benchmark collection does not contain examples like $\mathcal{T}_{2\text{-}c\text{-}invs}$). However, enabling either *acceleration via eventual decrease* or *acceleration via eventual increase* resulted in a significant improvement. Interestingly, there are many examples that can be accelerated with either of these two techniques: When both of them were enabled, LoAT (exactly or approximately) accelerated 1482 loops. When one of them was enabled, it accelerated 1444 resp. 1338 loops. But when none of them was enabled, it only accelerated 845 loops. We believe that this is due to examples like

$$\textbf{while } x_1 > 0 \wedge \ldots \textbf{ do } \begin{pmatrix} x_1 \\ x_2 \\ \ldots \end{pmatrix} \leftarrow \begin{pmatrix} x_2 \\ x_2 \\ \ldots \end{pmatrix}$$

where Thm. 11 *and* Thm. 13 are applicable (since $x_1 \leq x_2$ implies $x_2 \leq x_2$ and $x_1 \geq x_2$ implies $x_2 \geq x_2$).

Flata exactly accelerated 49 loops where LoAT failed or approximated and LoAT exactly accelerated 262 loops where Flata failed. So there were only 18 loops where both Flata and the full version of our calculus failed to compute an exact result. Among them were the only 3 examples where our implementation found a closed form, but failed anyway. One of them was[4]

[3] While acceleration via metering functions may be exact in some cases (see the discussion after Thm. 4), our implementation cannot check whether this is the case.

[4] The other two are structurally similar, but more complex.

$$\textbf{while } x_3 > 0 \textbf{ do } \begin{pmatrix} x_1 \\ x_2 \\ x_3 \end{pmatrix} \leftarrow \begin{pmatrix} x_1+1 \\ x_2-x_1 \\ x_3+x_2 \end{pmatrix}.$$

Here, the updated value of $x_1$ depends on $x_1$, the update of $x_2$ depends on $x_1$ and $x_2$, and the update of $x_3$ depends on $x_2$ and $x_3$. Hence, the closed form of $x_1$ is linear, the closed form of $x_2$ is quadratic, and the closed form of $x_3$ is cubic:

$$x_3^{(n)} = -\frac{1}{6} \cdot n^3 + \frac{1-x_1}{2} \cdot n^2 + \left(\frac{x_1}{2} + x_2 - \frac{1}{3}\right) \cdot n + x_3$$

So when fixing $x_1, x_2$, and $x_3$, $x_3^{(n)}$ has up to 2 extrema, i.e., its monotonicity may change twice. However, our techniques based on eventual monotonicity require that the respective expressions behave monotonically once they start to de- or increase, so these techniques only allow one change of monotonicity.

This raises the question if our approach can accelerate *every* loop with conjunctive guard and linear arithmetic whose closed form is a vector of (at most) quadratic polynomials with rational coefficients. We leave this to future work.

For our benchmark collection, links to the StarExec-jobs of our evaluation, and a pre-compiled binary (Linux, 64 bit) we refer to [14].

### 9 Conclusion and Future Work

After discussing existing acceleration techniques (Sec. 3), we presented a calculus to combine acceleration techniques modularly (Sec. 4). Then we showed how to combine existing (Sec. 5) and two novel (Sec. 6) acceleration techniques with our calculus. This improves over prior approaches, where acceleration techniques were used independently, and may thus improve acceleration-based verification techniques [6,7,11,16–18,28] in the future. An empirical evaluation (Sec. 8) shows that our approach is more powerful than state-of-the-art acceleration techniques. Moreover, if it is able to accelerate a loop, then the result is exact (instead of just an under-approximation) in most cases. Thus, our calculus can be used for under-approximating techniques (e.g., to find bugs or counterexamples) as well as in over-approximating settings (e.g., to prove safety or termination).

In the future, we plan to implement the missing features mentioned in Sec. 8 and integrate our novel calculus into our own acceleration-based program analyses to prove lower bounds on the runtime complexity [16,17] and non-termination [11] of integer programs. Furthermore, our experiments indicate that integrating specialized techniques for FMATs (cf. Sec. 7) would improve the power of our approach, as Flata exactly accelerated 49 loops where LoAT failed to do so (cf. Sec. 8). Moreover, we plan to design a *loop acceleration library*, such that our technique can easily be incorporated by other verification tools.

**Data Availability Statement and Acknowledgments** The tools and datasets used for the current study are available in the Zenodo repository [12].

I thank Carsten Fuhs, Marcel Hark, Sophie Tourret, and the anonymous reviewers for helpful feedback and comments. Moreover, I thank Radu Iosif and Filip Konecný for their help with Flata.
